# Sociodemographic and Clinical Characteristics Associated With Veterans’ Digital Needs

**DOI:** 10.1001/jamanetworkopen.2024.45327

**Published:** 2024-11-15

**Authors:** Lauren E. Russell, Portia Y. Cornell, Christopher W. Halladay, Meaghan A. Kennedy, Andrea Berkheimer, Emily Drucker, Leonie Heyworth, Sarah M. Leder, Kathleen M. Mitchell, Ernest Moy, Jennifer W. Silva, Brittany L. Trabaris, Lisa E. Wootton, Alicia J. Cohen

**Affiliations:** 1 Office of Health Equity, Veterans Health Administration, Washington, DC; 2 Center of Innovation in Transformative Health Systems Research to Improve Veteran Equity and Independence (THRIVE COIN), Providence, Rhode Island; 3 Centre for Digital Transformation of Health, University of Melbourne, Carlton, Victoria, Australia; 4 Centre for Health Policy, School of Global and Population Health, University of Melbourne, Carlton, Victoria, Australia; 5 New England Geriatric Research, Education, and Clinical Center, VA Bedford Healthcare System, Bedford, Massachusetts; 6 Department of Family Medicine, Boston University Chobanian and Avedisian School of Medicine, Boston, Massachusetts; 7 National Social Work Program, Care Management and Social Work Services, Veterans Health Administration, Washington, DC; 8 James E. Van Zandt Veterans Administration Medical Center, Altoona, Pennsylvania; 9 Office of Connected Care, Veterans Health Administration, Washington, DC; 10 Edward Hines Jr Veterans Administration Hospital, Hines, Illinois; 11 Department of Family Medicine, Alpert Medical School of Brown University, Providence, Rhode Island; 12 Department of Health Services, Policy and Practice, Brown University, Providence, Rhode Island

## Abstract

**Importance:**

Telehealth can expand access to care, but digital needs present barriers for some patients.

**Objective:**

To investigate sociodemographic and clinical associations of digital needs among veterans.

**Design, Setting, and Participants:**

This quality improvement study used data collected between July 2021 and September 2023 from Assessing Circumstances and Offering Resources for Needs (ACORN), a Department of Veterans Affairs (VA) initiative to systematically screen for, comprehensively assess, and address social risks and social needs. Eligible participants were veterans screened for social risks and social needs during routine care at 12 outpatient clinics, 3 emergency departments, and 1 inpatient unit across 14 VA medical centers. Data analysis occurred between October 2023 and January 2024.

**Exposure:**

The ACORN screening tool was administered by clinical staff.

**Main Outcomes and Measures:**

Veterans were considered positive for a digital need if they reported no smartphone or computer, no access to affordable and reliable internet, running out of minutes and/or data before the end of the month, and/or requested help setting up a video telehealth visit.

**Results:**

Among 6419 veterans screened (mean [SD] age, 67.6 [15.9] years; 716 female [11.2%]; 1740 Black or African American [27.1%]; 202 Hispanic or Latino [3.1%]; 4125 White [64.3%]), 2740 (42.7%) reported 1 or more digital needs. Adjusting for sociodemographic and clinical characteristics, the adjusted prevalence (AP) of lacking a device among veterans aged 80 years or older was 30.8% (95% CI, 27.9%-33.7%), 17.9% (95% CI, 16.5%-19.2%) among veterans aged 65 to 79 years, 9.9% (95% CI, 8.2%-11.6%) among veterans aged 50 to 64 years, 3.4% (95% CI, 2.1%-4.6%) among veterans aged 18 to 49 years, 17.6% (95% CI, 16.7%-18.6%) for males, and 7.9% (95% CI, 5.5%-10.3%) for females. AP of lacking affordable or reliable internet was 25.3% (95% CI, 22.6%-27.9%) among veterans aged 80 years or older, 15.0% (95% CI, 12.1%-18.0%) among veterans aged 18 to 49 years, 31.1% (95% CI, 28.9%-33.4%) for Black or African American veterans, 32.1% (95% CI, 25.2%-39.0%) for veterans belonging to other racial groups (ie, American Indian or Alaska Native, Asian, Native Hawaiian or Other Pacific Islander, as well as those with more than 1 race captured in their medical record), and 19.4% (95% CI, 18.2%-20.6%) for White veterans. Veterans with dementia were at higher risk of lacking a device (adjusted relative risk [aRR], 1.21; 95% CI, 1.00-1.48). Veterans with high medical complexity were at higher risk of lacking internet (aRR, 1.26; 95% CI, 1.11-1.42). Veterans with dementia (aRR, 1.58; 95% CI, 1.24-2.01) or substance use disorder (aRR, 1.22; 95% CI, 1.00-1.49) were more likely to want help scheduling a telehealth visit than those without.

**Conclusions and Relevance:**

In this quality improvement study of veterans screened for social risks and social needs, there were substantial disparities in digital needs. These findings suggest that routine screening is important to understand patients’ digital access barriers and connect patients with telehealth resources to address inequities in health care.

## Key Points

**Question:**

What sociodemographic characteristics and health conditions are associated with digital needs among veterans?

**Findings:**

In this quality improvement study with a cohort of 6419 veterans, digital needs questions integrated into a Department of Veterans Affairs social risk screening tool found that 42.7% of veterans screened reported 1 or more digital needs. There were substantial differences in digital needs associated with age, birth sex, race, income, and clinical complexity.

**Meaning:**

These findings suggest that routine screening and additional interventions to address the digital divide may be warranted to ensure equitable access to care.

## Teaser

This quality improvement study analyzes associations of sociodemographic and clinical factors with digital needs among individuals receiving care through the Veterans Health Administration.

## Introduction

Telehealth can improve access to both routine and nonemergent acute care by helping patients overcome social and geographic barriers to seeking in-person care.^1^ The COVID-19 pandemic accelerated the adoption of virtual care in the Department of Veterans Affairs (VA) health system.^2^ Even with resumption of nonemergency and elective in-person care after the initial months of the pandemic, telehealth has remained an integral aspect of care delivery.^3^ While increased use of video telehealth can expand access, it may also exacerbate inequities for patients without video-capable devices, access to the internet, or with low digital health literacy—each of which are necessary to engage with telehealth.^3,4,5,6^

In August 2020, the VA implemented a national Digital Divide Consult aimed to facilitate access to a device and internet connectivity and enhance access to care for veterans in geographically remote areas or with other barriers to in-person care who would benefit from telehealth.^7,8^ Through this initiative, veterans without a video-capable device or without reliable internet access receive a VA-loaned internet-connected device or assistance applying for federal internet subsidies based on a referral from their care team. However, the VA lacked a systematic way to identify veterans with digital needs. To address this gap, digital needs questions were developed and integrated into an existing VA initiative to systematically screen for, comprehensively assess, and address social risks and social needs: Assessing Circumstances and Offering Resources for Needs (ACORN).^9,10,11^ The updated ACORN screening tool was administered by clinical staff in outpatient, inpatient, and emergency department (ED) settings. In this study, we conducted an analysis of a subset of screenings that were administered in primary care, specialty care, inpatient settings, and EDs at 11 urban and 3 rural VA medical centers across the country. We assessed the frequency of reported digital needs among veterans who completed ACORN screenings and examined the association of sociodemographic and clinical characteristics with digital needs.

## Methods

As a quality improvement initiative, this study was determined by the VA Providence Healthcare System institutional review board to not require regulatory review or informed consent. We followed the Standards for Quality Improvement Reporting Excellence (SQUIRE) reporting guideline.^12^

### Development of Digital Needs Screening Questions

We developed digital needs questions with input from an interprofessional team of primary care physicians, nurses, social workers, researchers, VA operational partners, and other subject matter experts. We iteratively refined the questions based on cognitive interviewing with 10 veterans using think-aloud techniques to confirm face and content validity. We then pilot tested the tool with 31 veterans receiving care at a primary care clinic for veterans experiencing homelessness or at a VA on-campus encampment, further refining the instrument and workflow based on feedback from veterans and frontline staff. The 5 digital needs questions assessed access to an internet-capable device, access to affordable and reliable internet, running out of phone minutes and/or data on a cellular device, interest in scheduling a telehealth visit, and need for help learning to use a device for telehealth. Final digital needs questions were incorporated into the ACORN screening tool. The eFigure in Supplement 1 includes the questions and response options as they appear in the electronic medical record to the person administering the screening tool.

### Data Sources

Responses to the ACORN screening tool, sociodemographic data, and clinical characteristics were obtained from the VA Corporate Data Warehouse. We excluded 3 observations where veterans had a death date in their medical record prior to the ACORN screening date and 28 with contradictory responses, such as smartphone and none in response to, “Do you have access to any of the following devices?” Most veterans were screened only once during the study period. For veterans screened more than once, we defined their index screening as their first positive screen, or if no positives, then the first screen—thus, each individual appeared in the sample once.^13^

### Population

We included veterans screened between July 2021 through September 2023. The ACORN screening tool updated with digital needs questions was initially piloted in primary care settings at 4 urban and rural sites (ie, VA medical centers) beginning in July 2021 and integrated into existing VA National Social Work Program clinical initiatives.^14,15^ Selection of these sites was based on high engagement with other national projects and geographic location, with 3 of the 4 sites providing care for predominantly rural veterans. Based on demonstrated feasibility and acceptability at these 4 sites, an additional 10 sites started using ACORN (Table 1). Sites included in our analyses had been screening for at least 6 months as of September 30, 2023, and had completed at least 100 total screens.

**Table 1.** Sites and Clinical Settings Included in Analytic Sample

Site No.^a^	Rurality classification	US census region	Clinical setting	Start date	No. of unique veterans screened^b^
1	Rural	Northeast	Primary care social work, peer support, mental health, and whole health	July 2021	1205
2	Rural	South	Primary care social work	August 2021	628
3	Urban	South	Primary care social work	August 2021	303
4	Urban	South	Primary care social work	August 2021	168
5	Urban	West	Primary care social work	May 2022	138
6	Urban	South	Emergency department	May 2022	1981
7	Urban	West	Emergency department	May 2022	122
8	Rural	Midwest	Primary care social work	May 2022	212
9	Urban	Midwest	Primary care social work, heart failure clinic, and mental health	June 2022	758
10	Urban	Midwest	Emergency department and primary care social work	June 2022	247
11	Urban	South	Geriatric primary care	June 2022	282
12	Urban	West	Primary care social work	September 2022	131
13	Urban	Midwest	Women’s health	March 2023	145
14	Urban	Northeast	Inpatient (medical) and mental health	March 2023	99^c^

^a^ Sites included in our analyses had been screening for at least 6 months as of October 1, 2023, and had completed at least 100 total screens.

^b^ Number of veterans screened is based on data collected between the listed start date and September 30, 2023.

^c^ Though site 14 had 100 total screens, 1 veteran had been screened twice, thus the total number of unique veterans screened was 99.

The settings in which ACORN was implemented varied by site and included primary care, specialty care (eg, mental health or heart failure), inpatient care, and the ED (Table 1). All individuals screened were veterans enrolled in VA health care. Social workers, nurses, or peer specialists administered ACORN as part of routine care. In this pilot, target populations ranged from general screening to screening focused on specific subgroups perceived to be at higher risk for experiencing social needs, such as older veterans or women veterans. Workflows were also informed by individual site priorities and staffing capacity.

### Primary Outcomes

We grouped positive responses into 4 primary outcomes. Each outcome indicated a digital need: (1) not having a smartphone or computer, (2) often or sometimes lacking affordable and reliable internet, (3) often or sometimes running out of phone minutes and/or data, and (4) wanting help setting up a video visit.

### Explanatory Variables

We examined the association of digital needs with sociodemographic and clinical characteristics. Sociodemographic characteristics included age, birth sex, race (Black or African American, White, and veterans belonging to other racial groups, including American Indian or Alaska Native, Asian, Native Hawaiian or Other Pacific Islander, and those reporting more than 1 race), Hispanic or Latino ethnicity, relationship status (married or partnered vs not married or partnered), rurality, and VA enrollment priority group. Our measure of race and ethnicity represents a social construct, used as a proxy to measure the influence of structural racism experienced by veterans in our study.^16^ Race and ethnicity in the VA electronic health record are entered by staff, and though primarily self-reported by the patient, may sometimes be recorded based on staff observation.^17,18^ Priority group refers to veterans’ eligibility for and cost-share associated with VA health benefits, as well as service-connected disability compensation.^19^ Because we hypothesized that low-income veterans would be more likely to experience digital needs, we collapsed these into 3 categories: (1) service-connected (VA groups 1-4), low-income and not service-connected (VA group 5), and (3) not low-income and not service-connected (VA groups 6-8).

We used *International Statistical Classification of Diseases and Related Health Problems, Tenth Revision (ICD-10)* codes to identify the following diagnoses in the 12 months prior to screening: diabetes, heart failure, hypertension, Alzheimer disease or dementia, mental health conditions, and substance use disorder (eTable 1 in Supplement 1). We also examined Veterans’ Care Assessment Needs (CAN) score, a VA-validated risk prediction model based on sociodemographic and medical characteristics as well as health care utilization. CAN scores in the 95th percentile or greater are predictive of high risk of hospitalization or mortality in the subsequent 90 days.^20^

### Statistical Analyses

All analyses were conducted using R version 4.1.2 64 bit (R Project for Statistical Computing). We report the frequency and percentage of positive responses to each item. For veteran characteristics, we describe sample means overall and stratified by veterans reporting 1 or more digital needs vs no digital needs. We calculated *P* values for bivariate differences in the 2 groups using independent 2-sample *t* tests.

For sociodemographic variables, we estimated multivariable logistic regression adjusting for clinical and sociodemographic covariates. Because logistic regression models produce coefficients that are problematic to compare across populations or specifications,^21^ we report the population average marginal predicted prevalence within each sociodemographic subgroup from the estimated logistic models, adjusted for all other covariates (referred to as adjusted prevalence [AP]) using the modmarg package in R.^22^ For clinical characteristics, we estimated Poisson models with robust standard errors and the same set of covariates to report the relative risk of the outcome associated with having a comorbidity, compared with not having the condition.^23^ A threshold of *P* < .05 was used for statistical significance, using the delta method from calculating average marginal effects compared with the reference group in logistic regressions and *z* test statistics in Poisson regressions. Data analysis occurred from October 2023 to January 2024.

## Results

Of the 6419 veterans screened, mean (SD) age was 67.6 (15.9) years, 716 (11.2%) were female, 1740 (27.1%) were Black or African American, 202 (3.1%) were Hispanic or Latino, and 4125 (64.3%) were White (Table 2). Overall, 2740 veterans (42.7%) screened positive for 1 or more digital needs.

**Table 2.** Characteristics of Veterans Screened for Digital Needs

Characteristics	Veterans screened for ACORN, No. (%)			*P* value
	Total (N = 6419)	Negative (n = 3679)	Positive (n = 2740)	
Age, mean (SD), y	67.6 (15.9)	64.9 (16.8)	71.3 (13.6)	<.001
Age, y				
18-49	920 (14.3)	712 (19.4)	208 (7.6)	<.001
50-64	1240 (19.3)	746 (20.3)	494 (18.0)	
65-79	3098 (48.3)	1694 (46.0)	1404 (51.2)	
≥80	1161 (18.1)	527 (14.3)	634 (23.1)	
Birth sex				
Female	716 (11.2)	533 (14.5)	183 (6.7)	<.001
Male	5703 (88.8)	3146 (85.5)	2557 (93.3)	
Race				
Black or African American	1740 (27.1)	860 (23.4)	880 (32.1)	<.001
White	4125 (64.3)	2492 (67.7)	1633 (59.6)	
Other race^a^	186 (2.9)	94 (2.6)	92 (3.4)	
Unknown or missing	368 (5.7)	233 (6.3)	135 (4.9)	
Ethnicity				
Hispanic or Latino	202 (3.1)	124 (3.4)	78 (2.8)	.01
Not Hispanic or Latino	5906 (92.0)	3348 (91.0)	2558 (93.4)	
Unknown or missing	311 (4.8)	207 (5.6)	104 (3.8)	
Relationship status				
Married or partnered	3724 (58.0)	2264 (61.5)	1460 (53.3)	<.001
Not married or partnered	2.695 (42.0)	1415 (38.5)	1280 (46.7)	
VA rurality^b^				
Rural	2396 (37.3)	1337 (36.3)	1059 (38.6)	<.001
Urban	3404 (53.0)	1897 (51.6)	1507 (55.0)	
Unknown or missing	619 (9.6)	445 (12.1)	174 (6.4)	
VA enrollment priority group^c^				
Service connected	3734 (58.2)	2257 (61.3)	1477 (53.9)	<.001
Low-income and not service-connected	1592 (24.8)	755 (20.5)	837 (30.5)	
Not low-income and not service-connected	1067 (16.6)	649 (17.6)	418 (15.3)	
Unknown or missing	26 (0.4)	18 (0.5)	8 (0.3)	
CAN score ≥95	2265 (35.3)	1149 (31.2)	1116 (40.7)	<.001
Smoking or tobacco use	1200 (18.7)	641 (17.4)	559 (20.4)	.003
Diabetes	2232 (34.8)	1246 (33.9)	986 (36.0)	.08
Heart failure	1162 (18.1)	615 (16.7)	547 (20.0)	.001
Hypertension	4082 (63.6)	2233 (60.7)	1849 (67.5)	<.001
Alzheimer disease or dementia	463 (7.2)	216 (5.9)	247 (9.0)	<.001
Mental health condition^d^	2825 (44.0)	1678 (45.6)	1147 (41.9)	.003
Substance use disorder	1038 (16.2)	534 (14.5)	504 (18.4)	<.001

Abbreviations: ACORN, Assessing Circumstances and Offering Resources for Needs; CAN, Care Assessment Needs; VA, Veterans Affairs.

^a^ Other race includes American Indian or Alaska Native, Asian, Native Hawaiian or Other Pacific Islander, as well as those with more than 1 race captured in their medical record.

^b^ Rurality refers to the veteran’s rurality based on the most recent address listed in their medical record. These data are based on the rural-urban commuting areas system developed by the US Department of Agriculture and the US Department of Health and Human Services. There are 3 categories reported: urban, rural, and highly rural. We combined rural and highly rural for our analyses.

^c^ Enrollment priority determines US veterans’ eligibility for and cost-share associated with VA health services. Enrollment priority group is collapsed into 3 categories: (1) veterans receiving some percentage of service-connected disability compensation, veterans receiving aid and attendance, or veterans having experienced a non–service-connected catastrophic disability (ie, groups 1-4 [service connected]); (2) veterans with no service-connected disability compensation who are low-income (ie, group 5 [low-income and not service connected]); and (3) those with no service-connected disability compensation who are not low-income (above the VA means test; ie, groups 6-8 [not low-income and not service-connected]).

^d^ See eTable 2 in Supplement 1 for a list of included conditions.

Of all veterans screened, 1082 (16.9%) reported not having a smartphone or computer, 1486 (23.2%) sometimes or often lacked affordable and reliable internet, and 187 (2.9%) sometimes or often ran out of phone minutes and/or data before the end of the month (Table 3). Of all veterans, 777 (12.1%) reported wanting help setting up a video visit or did not know what a video visit was; among these veterans, 347 (44.7%) needed help learning to use a telehealth device.

**Table 3.** Responses to Digital Needs Screening Questions Among Veterans Screened

Question and response option	Responses, No. (%) (N = 6419)
Access to devices^a^	
Landline^b^	606 (9.4)
Simple cell phone (flip phone)	929 (14.5)
Smartphone (a cell phone with a touch screen and internet)	4780 (74.5)
Computer (laptop, desktop, or tablet)	2034 (31.7)
None	231 (3.6)
Veteran declined to answer	290 (4.5)
No smartphone or computer^c^	1082 (16.9)
Affordable and reliable internet	
Yes	3902 (60.8)
No	1486 (23.2)
I do not want internet access at home	535 (8.3)
Veteran declined to answer	489 (7.6)
Run out of phone minutes and/or data	
Often	57 (0.9)
Sometimes	130 (2.0)
Never	5495 (85.6)
I do not have a cell phone (flip phone or smartphone)	200 (3.1)
Veteran declined to answer	487 (7.6)
Wants help setting up a video visit	
Yes	601 (9.4)
No	2618 (40.8)
I already know how to do video visits/do not need help	2417 (37.7)
I do not know what a video visit is	176 (2.7)
Veteran declined to answer	599 (9.3)
Wants help learning to use a device^d^	
Yes	347 (44.7)
No	222 (28.6)
I do not have any of these devices	19 (2.4)
Veteran declined to answer	4 (0.5)

^a^ This question was asked as a check all that apply, so the percentages exceed 100%.

^b^ The landline option was added to the ACORN screening tool in September 2022 and approximately one-half of screenings included this option. Thus, this frequency is likely an underestimate of veterans with access to a landline.

^c^ Not an offered response option; the sum was calculated by the authors.

^d^ Only answered if veterans reported wanting help setting up a future video visit or not knowing what a video visit is to question 4 (777 participants).

**Figure 1.**
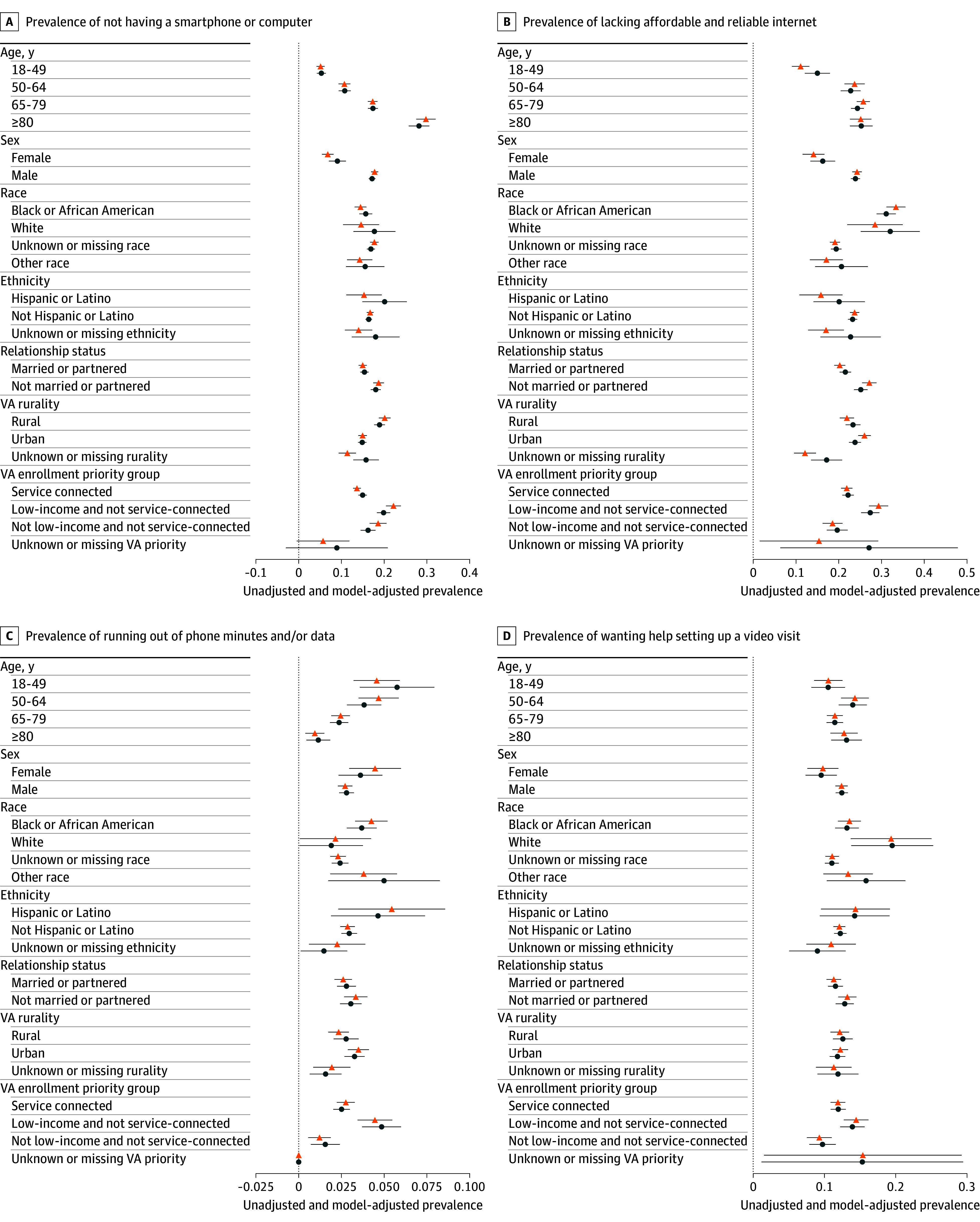
Adjusted Prevalence of Digital Needs Associated With Sociodemographic Characteristics Unadjusted and model-adjusted prevalence of digital needs, adjusting for all covariates (see Table 2 for the covariates and their definitions). Model-adjusted prevalences are the population-average estimated margins by subgroup from a multivariate logistic regression. Other race includes American Indian or Alaska Native, Asian, Native Hawaiian or Other Pacific Islander, as well as those with more than 1 race captured in their medical record. VA indicates Department of Veterans Affairs.

AP of individual digital needs by sociodemographic characteristics are shown in Figure 1. Full model estimates, AP, and marginal effects are reported in eTables 2 to 11 in Supplement 1. Adjusting for sociodemographic and clinical characteristics, the AP of having a smartphone or computer decreased with increasing age. Compared with veterans aged 18 to 49 years (AP, 3.4%; 95% CI, 2.1%-4.6%), veterans were significantly more likely to lack a smartphone or computer among those aged 50 to 64 years (AP, 9.9%; 95% CI, 8.2%-11.6%; adjusted odds ratio [aOR], 3.21; 95% CI, 2.08-4.94), 65 to 79 years (AP, 17.9%; 95% CI, 16.5%-19.2%; aOR, 6.42; 95% CI, 4.25-9.69), and 80 years or older (AP, 30.8%; 95% CI, 27.9%-33.7%; aOR, 13.49; 95% CI, 8.77-20.75). Additionally, compared with veterans aged 18 to 49 years (AP, 15.0%; 95% CI, 12.1%-18.0%), veterans were also more likely to lack affordable and reliable internet among those aged 50 to 64 years (AP, 22.8%; 95% CI, 20.5%-25.1%; aOR, 1.72; 95% CI, 1.31-2.23), 65 to 79 years (AP, 24.4%; 95% CI, 22.9%-25.9%; aOR, 1.87; 95% CI, 1.44-2.43), and 80 years or older (AP, 25.3%; 95% CI, 22.6%-27.9%; aOR, 1.97; 95% CI, 1.47-2.64). Compared with veterans aged 18 to 49 years (AP, 5.8%; 95% CI, 3.6%-7.9%) veterans were significantly less likely to run out of phone minutes and/or data among those aged 65 to 79 years (AP, 2.4%; 95% CI, 1.8%-2.9%; aOR, 0.39; 95% CI, 0.24-0.64) and 80 years or older (AP, 1.2%; 95% CI, 0.5%-1.8%; aOR, 0.19; 95% CI, 0.09-0.40). Veterans aged 50 to 64 years (AP, 14.0%; 95% CI, 12.0%-15.9%) were significantly more likely to want help setting up a video visit than those aged 18 to 49 years (AP, 10.5%; 95% CI, 8.2%-12.9%; aOR, 1.39; 95% CI, 1.03-1.86). The AP of lacking a device was 17.6% (95% CI, 16.7%-18.6%) for males and 7.9% (95% CI, 5.5%-10.3%) for females. Compared with males (AP, 23.9%; 95% CI, 22.8%-25.0%), females were less likely to lack affordable and reliable internet (AP, 16.3%; 95% CI, 13.4%-19.2%; aOR, 0.60; 95% CI, 0.48-0.76).

Adjusting for sociodemographic and clinical characteristics, digital needs differed across racial groups. Compared with White veterans (AP, 19.4%; 95% CI, 18.2%-20.6%), there was a higher probability of lacking affordable and reliable internet among Black or African American veterans (AP, 31.1%; 95% CI, 28.9%-33.4%; aOR, 1.92; 95% CI, 1.67-2.20) and veterans belonging to other racial groups (AP, 32.1%; 95% CI, 25.2%-39.0%; aOR, 2.01; 95% CI, 1.43-2.83). Compared with White veterans (AP, 2.4%; 95% CI, 1.9%-2.9%), Black or African American veterans were also more likely to run out of phone minutes and/or data (AP, 3.7%; 95% CI, 2.8%-4.6%; aOR, 1.56; 95% CI, 1.11-2.18). Compared with White veterans (AP, 11.0%; 95% CI, 10.1%-12.0%), there was a higher likelihood of wanting help setting up a video visit among Black or African American veterans (AP, 13.2%; 95% CI, 11.5%-14.8%; aOR, 1.22; 95% CI, 1.02-1.47) and veterans belonging to other racial groups (AP, 19.5%; 95% CI, 13.8%-25.3%; aOR, 1.97; 95% CI, 1.34-2.89). We did not find statistically significant differences in any outcome by Hispanic or Latino ethnicity.

Compared with veterans who were not married or partnered (AP, 18.6%; 95% CI, 17.2%-20.1%), veterans who were married or partnered (AP, 15.5%; 95% CI, 14.3%-16.6%) were significantly less likely to lack a smartphone or computer (aOR, 0.78; 95% CI, 0.68-0.90). Additionally, compared with veterans who were not married or partnered (AP, 25.2%; 95% CI, 23.6%-26.8%), veterans who were married or partnered (AP, 21.6%; 95% CI, 20.2%-22.9%) were less likely to lack and affordable and reliable internet (aOR, 0.81; 95% CI, 0.71-0.91). Veterans in rural areas (AP, 19.7%; 95% CI, 18.2%-21.2%) were significantly more likely to report not having a smartphone or computer compared with veterans in urban areas (AP, 14.9%; 95% CI, 13.7%-16.0%; aOR, 1.45; 95% CI, 1.25-1.68).

Veterans who were low-income and not service-connected were significantly more likely to not have a smartphone or computer, lack affordable and reliable internet, and run out of phone minutes and/or data compared with service-connected veterans and veterans who were not low-income and not service-connected (eTables 2-4 in Supplement 1). Compared with veterans who were low-income and not service-connected (AP, 13.9%; 95% CI, 12.2%-15.6%), veterans who were not low-income and not service-connected were less likely to request help setting up a video telehealth visit (AP, 9.7%; 95% CI, 7.9%-11.6%; aOR, 0.66; 95% CI, 0.51-0.86).

**Figure 2.**
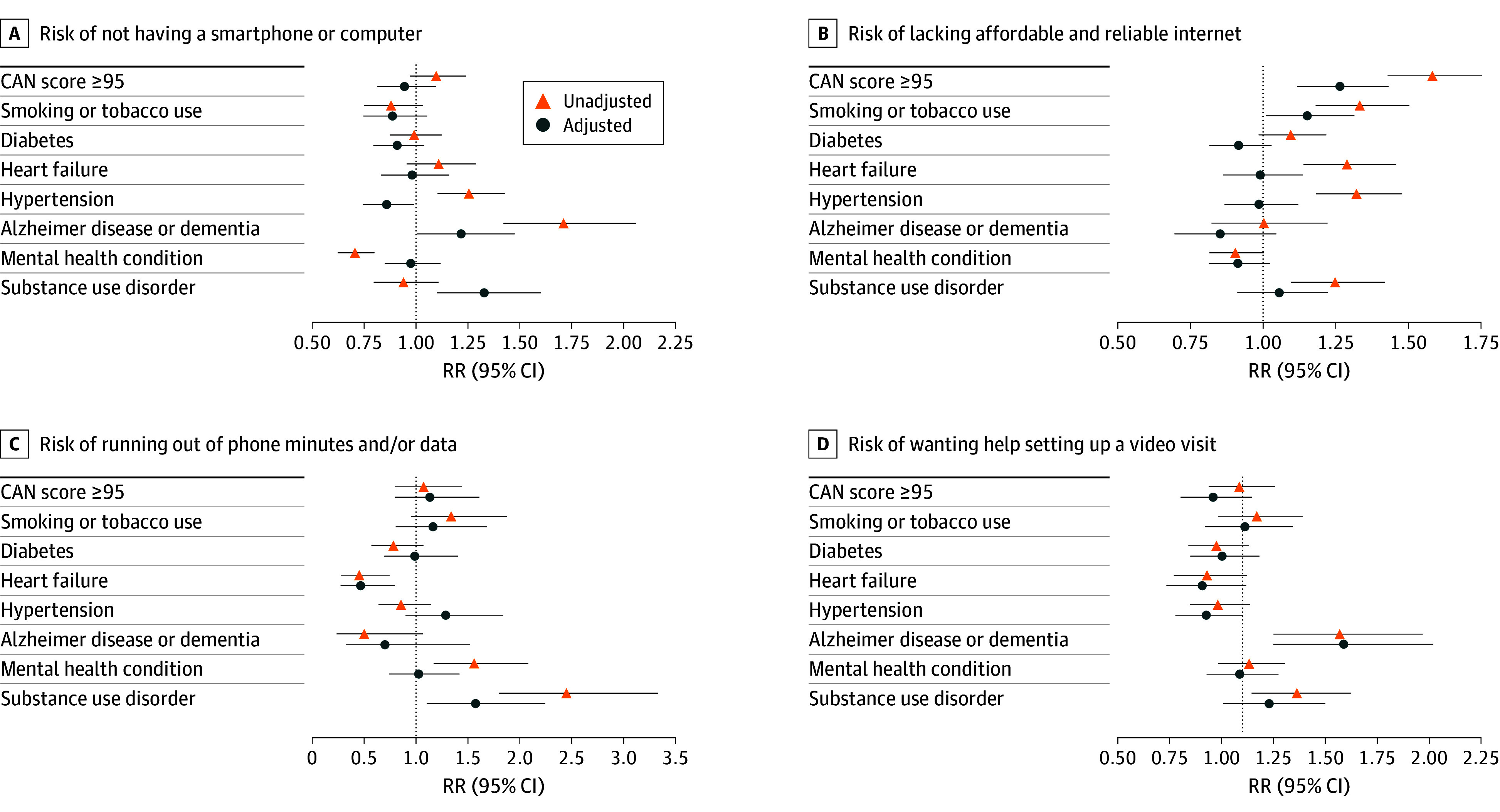
Relative Risk of Digital Needs Associated With Clinical Characteristics Poisson models were used to estimate relative risks. Adjusted models control for covariates listed in Table 2. CAN indicates Care Assessment Needs; RR, relative risk.

Figure 2 shows relative risk (RR) for individual digital needs associated with clinical characteristics. Adjusting for sociodemographic and clinical characteristics, veterans with a CAN score of 95 or greater were more likely to lack affordable and reliable internet compared with those with a CAN score lower than 95 (adjusted RR [aRR], 1.26; 95% CI, 1.11-1.42). Veterans with Alzheimer disease or dementia had a higher risk of reporting no smartphone or computer (aRR, 1.21; 95% CI, 1.00-1.48) and were more likely to want help setting up a video visit (aRR, 1.58; 95% CI, 1.24-2.01), compared with veterans without those diagnoses. Veterans with substance use disorder were more likely to lack a smartphone or computer (aRR, 1.33; 95% CI, 1.10-1.60), more likely to run out of phone minutes or data (aRR, 1.57; 95% CI, 1.10-2.25), and more likely to want help setting up a video visit (aRR, 1.22; 95% CI, 1.00-1.49), compared with veterans without substance use disorder. Veterans with heart failure were less likely to run out of phone minutes and/or data compared to veterans without heart failure (aRR, 0.45; 95% CI, 0.28-0.75). Veterans who were current smokers were more likely to lack affordable and reliable internet than those who were not current smokers (aRR, 1.33; 95% CI, 1.18-1.49). Diabetes, hypertension, and mental health conditions were not associated with probability of endorsing any digital needs.

## Discussion

In this quality improvement study, more than 40% of veterans screened with ACORN reported at least 1 digital need. The most frequently reported need was often or sometimes lacking affordable and reliable internet. Our findings were consistent with previous work showing that older adults were more likely to experience digital needs.^24,25,26,27^ Our finding that older veterans were less likely to run out of phone minutes and/or data may be due to differences in device access because older veterans were also more likely to report not having a smartphone or computer. Similar to previous studies, Black or African American veterans and veterans belonging to other racial groups were more likely to report digital needs than White veterans,^28^ but our lack of significant differences across any digital need by ethnicity contrasts with other studies showing differences in access and use of telehealth among Hispanic and Latino compared with non-Hispanic and Latino populations.^28,29,30^

Our findings have the potential to improve clinical practice and policy by highlighting populations at higher risk for digital inequities and who may qualify for VA or community resources to address digital needs. Veterans who were older, not married or partnered, and low-income were also among those at highest risk for digital needs, which may exacerbate geographic and financial barriers to in-person care (eg, lack of transportation or difficulty getting sufficient time off from work). Veterans with complex conditions such as heart failure or high CAN scores are targeted for focused care-coordination resources^31^; lack of internet access may present a barrier for some veterans to receiving the supports they need. Given that dementia was associated with reporting a lack of devices and desire to set up a video visit, future work could investigate digital needs among veterans with caregivers because telehealth visits could improve caregivers’ communication with the care team. Additionally, it will be important to consider how digital inequities might prevent some veterans from accessing digital health tools. Relatedly, some rural veterans may have difficulty accessing digital tools or virtual care due to limited broadband options and connectivity challenges in rural areas of the US.^6^

The VA has robust resources to address digital needs, including the national Digital Divide Consult, which can be used to help veterans obtain a VA-loaned internet-connected device to connect with VA clinicians as well as support applying for federal internet subsidies. The VA also provides support for digital health literacy, including local telehealth technicians at VA medical centers and virtual health resource centers.^32^ Members of the VA interdisciplinary care team can refer or connect veterans to federal programs and national nonprofits that can provide assistance with digital access needs including Lifeline^33^ and EveryoneOn.^34^ The Affordable Connectivity Program^35^ was also previously an option; however, this program stopped accepting new applications in February 2024 and the last fully funded month of the program was April 2024.^36^ Improving internet accessibility remains a federal priority in the US. In June 2023, the Department of Commerce National Telecommunications and Information Administration announced the allocation of $42.45 billion for the Broadband Equity, Access, and Deployment program.^36^

Screening for digital needs as part of ACORN is ongoing. As of October 2024, the ACORN initiative has expanded to 60 VA medial centers nationally and is administered across an increasing range of clinical settings. This ongoing expansion will provide insight into how digital needs among veterans may differ by sociodemographic characteristics, clinical settings, and programs. As part of ACORN workflows, veterans who endorse digital needs may be provided with geographically tailored resource guides and/or support navigating VA and community resources such as VA virtual health resource centers and EveryoneOn. When appropriate, veterans are referred to social workers for further assessment and follow-up, including eligibility for the Digital Divide Consult. Future research is needed to assess the effectiveness of this screening and the provision of resources and referrals in addressing identified digital needs. Optimal practices for screening may vary based on patient preferences and needs, available resources (eg, staffing), and technological infrastructure. In addition to adapting and tailoring ACORN to a variety of clinical settings and programs, there is a need to develop a systematic follow-up process that captures whether veterans’ needs have been addressed.

### Limitations

This study has several limitations. More than one-half of the VA medical centers included in the analysis conducted screening among veterans who had already been referred to social workers through primary care, and therefore may not be representative of the broader VA population. Additionally, each site developed their own workflow for integrating ACORN into clinical care including which veterans to screen, and staff used clinical judgement to determine situations in which screening was not appropriate. Because clinical staff may have been more likely to screen veterans experiencing social distress, our findings are not estimates of the overall prevalence of digital needs in the VA population. However, any bias in the correlation (prevalence difference) associated with other characteristics is difficult to speculate. Furthermore, the number and sociodemographic characteristics of veterans declining screening or for whom screening was otherwise not administered were not reliably collected by clinic staff. Responses were self-reported, and findings may also be susceptible to social desirability bias, although questions were asked by trained clinical staff and contextualized as a means of being able to connect veterans with needed resources.

## Conclusions

In this quality improvement study of veterans screened for social needs, more than 40% endorsed digital needs. This study identified associations of veterans’ digital needs with older age, male birth sex, rural residence, low income, nonmarried or nonpartnered status, minoritized race, complex medical needs, dementia, and substance use. The inclusion of digital needs questions in a broader social risks and social needs clinical screening tool can help to systematically identify needs among these populations that may otherwise go undetected. Screening for digital needs in clinical settings is an essential step toward connecting patients with resources needed to engage in telehealth, and overcoming social, physical, and geographic barriers that may otherwise hinder veterans’ access to health care.

## Notes

Supplement 1. **eFigure.** Digital Divide Screening Questions and Response Options **eTable 1**. ICD-10 Codes Used for Clinical Characteristics **eTable 2.** Sociodemographic Correlates of Screening Positive for Not Having a Smart Phone or a Computer **eTable 3.** Sociodemographic Correlates of Screening Positive for Not Having Affordable or Reliable Internet **eTable 4.** Sociodemographic Correlates of Screening Positive for Running Out of Minutes Sometimes or Often **eTable 5**. Sociodemographic Correlates of Screening Positive for Requesting Assistance in Setting Up a Video Telehealth Encounter **eTable 6.** Logistic Model Predicted Margins and 95% Confidence Intervals (Without Smartphone or Computer; Without Affordable and Reliable Internet) **eTable 7.** Logistic Model Predicted Margins and 95% Confidence Intervals (Run Out of Phone Minutes or Data; Want Help With a Video Or Telehealth Visit) **eTable 8.** Poisson Models of Association of Clinical Conditions and Being Without Smartphone or Computer **eTable 9.** Poisson Models of Association of Clinical Conditions and Being Without Affordable and Reliable Internet **eTable 10.** Poisson Models of Association of Clinical Conditions and Running Out of Phone Minutes or Data **eTable 11.** Poisson Models of Association of Clinical Conditions and Wanting Help With a Video Telehealth Visit

Supplement 2.
Data Sharing Statement
