# Supplementary material for: Sociodemographic and Clinical Characteristics Associated With Veterans’ Digital Needs
Source: JAMA Netw Open. 2024 Nov 15;7(11):e2445327. doi: 10.1001/jamanetworkopen.2024.45327 (PMC11568462; doi:10.1001/jamanetworkopen.2024.45327)
Supplement: Supplement 1. — eFigure. Digital Divide Screening Questions and Response Options eTable 1. ICD-10 Codes Used for Clinical Characteristics eTable 2. Sociodemographic Correlates of Screening Positive for Not Having a Smart Phone or a Computer eTable 3. Sociodemographic Correlates of Screening Positive for Not Having Affordable or Reliable Internet eTable 4. Sociodemographic Correlates of Screening Positive for Running Out of Minutes Sometimes or Often eTable 5. Sociodemographic Correlates of Screening Positive for Requesting Assistance in Setting Up a Video Telehealth Encounter eTable 6. Logistic Model Predicted Margins and 95% Confidence Intervals (Without Smartphone or Computer; Without Affordable and Reliable Internet) eTable 7. Logistic Model Predicted Margins and 95% Confidence Intervals (Run Out of Phone Minutes or Data; Want Help With a Video Or Telehealth Visit) eTable 8. Poisson Models of Association of Clinical Conditions and Being Without Smartphone or Computer eTable 9. Poisson Models of Association of Clinical Conditions and Being Without Affordable and Reliable Internet eTable 10. Poisson Models of Association of Clinical Conditions and Running Out of Phone Minutes or Data eTable 11. Poisson Models of Association of Clinical Conditions and Wanting Help With a Video Telehealth Visit [file jamanetwopen-e2445327-s001.pdf]

Supplemental Online Content

Russell LE, Cornell PY, Halladay CW, et al. Sociodemographic and clinical characteristics associated with veterans’ digital needs. *JAMA Netw. Open*. 2024;7(11):e2445327. doi:10.1001/jamanetworkopen.2024.45327

eFigure. Digital Divide Screening Questions and Response Options
eTable 1. *ICD-10* Codes Used for Clinical Characteristics
eTable 2. Sociodemographic Correlates of Screening Positive for Not Having a Smart Phone or a Computer
eTable 3. Sociodemographic Correlates of Screening Positive for Not Having Affordable or Reliable Internet
eTable 4. Sociodemographic Correlates of Screening Positive for Running Out of Minutes Sometimes or Often
eTable 5. Sociodemographic Correlates of Screening Positive for Requesting Assistance in Setting Up a Video Telehealth Encounter
eTable 6. Logistic Model Predicted Margins and 95% Confidence Intervals (Without Smartphone or Computer; Without Affordable and Reliable Internet)
eTable 7. Logistic Model Predicted Margins and 95% Confidence Intervals (Run Out of Phone Minutes or Data; Want Help With a Video Or Telehealth Visit)
eTable 8. Poisson Models of Association of Clinical Conditions and Being Without Smartphone or Computer
eTable 9. Poisson Models of Association of Clinical Conditions and Being Without Affordable and Reliable Internet
eTable 10. Poisson Models of Association of Clinical Conditions and Running Out of Phone Minutes or Data
eTable 11. Poisson Models of Association of Clinical Conditions and Wanting Help With a Video Telehealth Visit

This supplemental material has been provided by the authors to give readers additional information about their work.

© 2024 Russell LE et al. *JAMA Network Open.*

eFigure. Digital Divide Screening Questions and Response Options

Do you have access to any of the following devices? (Please select all that apply)

- ☐ Landline
- ☐ Simple cell phone (flip phone)
- ☐ Smartphone (a cell phone with a touch screen and internet)
- ☐ Computer (laptop, desktop, or tablet such as an iPad)
- ☐ None
- ☐ Veteran declined to answer

Do you have access to affordable and reliable internet at home?

- ○ Yes
- ○ No
- ○ I don't want internet access at home
- ○ Veteran declined to answer

How often do you run out of phone minutes and/or data before the end of the month?

- ○ Often
- ○ Sometimes
- ○ Never
- ○ I don't have a cell phone (flip phone or smartphone)
- ○ Veteran declined to answer

Veterans can now have video visits with their care team from home or another location of their choice. Would you like help setting up a future video visit with a member of your VA care team?

- ◉ Yes
  - Do you need help learning to use a smart phone, tablet, or computer for video visits at the VA?
    - ○ Yes
    - ○ No
    - ○ I don't have any of these devices
    - ○ Veteran declined to answer
- ○ No
- ○ I already know how to do video visits/don't need help
- ○ I don't know what a video visit is
- ○ Veteran declined to answer

**Disposition / Action Steps**	Follow-up post-screener: Ask the Veteran if they would like assistance with any of the above needs.	Digital Divide Program (w/ optional text box)[a]<br>Warm hand-off/connection<br>Consult (for ordering clinicians only)

Notes: Sample includes all Veterans given the ACORN screener (answered at least 1 ACORN screening question).

a. Person administering screener must select Digital Divide Program as an action step to see response options below

b. Veterans who have "none," a landline or flip phone but no smartphone or computer, were considered positive for digital need.

c. The "landline" option was added to the screener in September 2022 and approximately half of screenings included this option.

© 2024 Russell LE et al. *JAMA Network Open.*

eTable 1. ***ICD-10*** **Codes Used for Clinical Characteristics**

Study Measure	Diagnosis Category	ICD-10 codes
Alzheimer’s or Dementia	Atypical virus infections of central nervous system	A81., A81.0, A81.00, A81.01, A81.09, A81.2, A81.82, A81.89, A81.9
	Vascular dementia	F01.50, F01.51
	Dementia in other diseases classified elsewhere	F02.80, F02.81
	Unspecified dementia	F03.90, F03.91
	Amnestic Disorder, delirium, catatonic, and other specified mental disorders (due to known physiological condition)	F06.1, F06.8
	Systemic atrophy primarily affecting central nervous system in other diseases classified elsewhere	G13.8
	Alzheimer's disease	G30.0, G30.1, G30.8, G30.9
	Pick's disease	G31.01
	Other frontotemporal dementia; Other frontotemporal neurocognitive disorder	G31.09
	Senile degeneration of brain, not elsewhere classified	G31.1
	Degeneration of nervous system due to alcohol	G31.2
	Other disorders of brain in diseases classified elsewhere	G94.0
	Age-related cognitive decline or physical disability	R41.81, R54.0
Tobacco/Smoking Use	Nicotine dependence	F17.200, F17.201, F17.203, F17.208, F17.209, F17.210, F17.211, F17.213, F17.218, F17.219, F17.220, F17.221, F17.223, F17.228, F17.229, F17.290, F17.291, F17.293, F17.298, F17.299
	Simple chronic bronchitis	J41.0

© 2024 Russell LE et al. *JAMA Network Open.*

Study Measure	Diagnosis Category	ICD-10 codes
	Personal history of nicotine dependence	Z87.891
Diabetes	Diabetes mellitus due to underlying condition	E08.00, E08.01. E08.10, E08.11, E08.21, E08.22, E08.29, E08.311, E08.319. E08.321, E08.329, E08.331, E08.339, E08.341, E08.349, E08.351, E08.359, E08.36, E08.39, E08.40, E08.41, E08.42, E08.43, E08.44, E08.49, E08.51, E08.52, E08.59, E08.610, E08.618, E08.620, E08.621, E08.622, E08.628, E08.630, E08.638, E08.641, E08.649, E08.65, E08.69, E08.8, E08.9
	Drug or chemical induced diabetes mellitus	E09.00, E09.01, E09.10, E09.11, E09.21, E09.22, E09.29, E09.311, E09.319, E09.321, E09.329, E09.331, E09.339, E09.341, E09.349, E09.351, E09.359, E09.36, E09.39, E09.40, E09.41, E09.42, E09.43, E09.44, E09.49, E09.51, E09.52, E09.59, E09.610, E09.618, E09.620, E09.621, E09.622, E09.628, E09.630, E09.638, E09.641, E09.649, E09.65, E09.69, E09.8, E09.9
	Type 1 diabetes mellitus	E10.10, E10.11, E10.21, E10.22, E10.29, E10.311, E10.319, E10.321, E10.329, E10.331, E10.339, E10.341, E10.349, E10.351, E10.359, E10.36, E10.39, E10.40, E10.41, E10.42, E10.43, E10.44, E10.49, E10.51, E10.52, E10.59, E10.610, E10.618, E10.620, E10.621, E10.622, E10.628, E10.630, E10.638, E10.641, E10.649, E10.65, E10.69, E10.8, E10.9
	Type 2 diabetes mellitus	E11.00, E11.01, E11.21, E11.22, E11.29, E11.311, E11.319, E11.321, E11.329, E11.331, E11.339, E11.341, E11.349, E11.351, E11.359, E11.36, E11.39, E11.40, E11.41, E11.42, E11.43, E11.44, E11.49, E11.51, E11.52, E11.59, E11.610, E11.618, E11.620, E11.621, E11.622, E11.628, E11.630, E11.638, E11.641, E11.649, E11.65, E11.69, E11.8, E11.9
	Other specified diabetes mellitus	E13.00, E13.01, E13.10, E13.11, E13.21, E13.22, E13.29, E13.311, E13.319, E13.321, E13.329, E13.331, E13.339, E13.341, E13.349, E13.351, E13.359, E13.36, E13.39, E13.40, E13.41, E13.42, E13.43, E13.44, E13.49, E13.51, E13.52, E13.59, E13.610, E13.618, E13.620, E13.621, E13.622, E13.628, E13.630, E13.638, E13.641, E13.649, E13.65, E13.69, E13.8, E13.9
	Pre-existing diabetes mellitus	O24.011, O24.012, O24.013, O24.019, O24.02, O24.03, O24.111, O24.112, O24.113, O24.119, O24.12, O24.13, O24.311, O24.312, O24.313, O24.319, O24.32, O24.33, O24.811, O24.812, O24.813, O24.819, O24.82, O24.83
	Unspecified diabetes mellitus in pregnancy, childbirth, or puerperium	O24.911, O24.912, O24.913, O24.92, O24.93

© 2024 Russell LE et al. *JAMA Network Open.*

Study Measure	Diagnosis Category	ICD-10 codes
Heart Failure	Rheumatic heart failure	I09.81
	Hypertensive heart disease with heart failure	I11.0
	Hypertensive heart and chronic kidney disease with heart failure	I13.0, I13.2
	Left ventricular failure	I50.1
	Systolic (congestive) heart failure, including unspecified, acute, and chronic	I50.20, I50.21, I50.22
	Acute on chronic systolic (congestive) heart failure	I50.23
	Diastolic (congestive) heart failure, including unspecified, acute, and chronic	I50.30, I50.31, I50.32
	Acute on chronic diastolic (congestive) heart failure	I50.33
	Combined systolic (congestive) and diastolic (congestive) heart failure, including unspecified, acute, and chronic	I50.40, I50.41, I50.42
	Acute on chronic combined systolic (congestive) and diastolic (congestive) heart failure	I50.43
	Heart failure, unspecified	I50.9
Hypertension	Hypertensive retinopathy	H35.031, H35.032, H35.033, H35.039
	Essential (primary) Hypertension	I10.0
	Hypertensive heart disease	I11.0, I11.9
	Hypertensive chronic kidney disease	I12.0, I12.9
	Hypertensive heart and chronic kidney disease	I13.0, I13.10, I13.11, I13.2

© 2024 Russell LE et al. *JAMA Network Open.*

Study Measure	Diagnosis Category	ICD-10 codes
	Renovascular hypertension	I15.0
	Secondary hypertension	I15.1, I15.2, I15.8, I15.9
	Primary pulmonary hypertension	I27.0
	Hypertensive encephalopathy	I67.4
Substance Use Disorder	Alcohol related disorders	F10.27, F19.97, F10.10, F10.120, F10.121, F10.129, F10.14, F10.150, F10.151, F10.159, F10.180, F10.181, F10.182, F10.188, F10.19, F10.20, F10.21, F10.220, F10.221, F10.229, F10.230, F10.231, F10.232, F10.239, F10.24, F10.250, F10.251, F10.259, F10.26, F10.280, F10.281, F10.282, F10.288, F10.29, F10.920, F10.921, F10.929, F10.94, F10.950, F10.951, F10.959, F10.96, F10.97, F10.980, F10.981, F10.982, F10.988, F10.99
	Opioid related disorders	F11.10, F11.120, F11.121, F11.122, F11.129, F11.14, F11.150, F11.151, F11.159, F11.181, F11.182, F11.188, F11.19, F11.20, F11.21, F11.220, F11.221, F11.222, F11.229, F11.23, F11.24, F11.250, F11.251, F11.259, F11.281, F11.282, F11.288, F11.29, F11.90, F11.920, F11.921, F11.922 F11.929, F11.93, F11.94, F11.950, F11.951, F11.959, F11.981, F11.982, F11.988, F11.99
	Cannabis related disorders	F12.10, F12.120, F12.121, F12.122, F12.129, F12.150, F12.151, F12.159, F12.180, F12.188, F12.19, F12.20, F12.21, F12.220, F12.221, F12.222, F12.229, F12.250, F12.251, F12.259, F12.280, F12.288, F12.29, F12.90, F12.920, F12.921, F12.922, F12.929, F12.950, F12.951, F12.959, F12.980, F12.988, F12.99
	Sedative, hypnotic, or anxiolytic related disorders	F13.10, F13.120, F13.121, F13.129, F13.14, F13.150, F13.151, F13.159, F13.180, F13.181, F13.182, F13.188, F13.19, F13.20, F13.21 F13.220, F13.221, F13.229, F13.230, F13.231, F13.232, F13.239, F13.24, F13.250, F13.251, F13.259, F13.26, F13.27, F13.280, F13.281, F13.282, F13.288, F13.29, F13.90, F13.920, F13.921, F13.929, F13.930, F13.931, F13.932, F13.939, F13.94, F13.950, F13.951, F13.959, F13.96, F13.97, F13.980, F13.981, F13.982, F13.988, F13.99
	Cocaine related disorders	F14.10, F14.120, F14.121, F14.122, F14.129, F14.14, F14.150, F14.151, F14.159, F14.180, F14.181, F14.182, F14.188,<br>F14.19, F14.20, F14.21, F14.220, F14.221, F14.222, F14.229, F14.23, F14.24, F14.250, F14.251, F14.259, F14.280, F14.281, F14.282, F14.288, F14.29, F14.90,

© 2024 Russell LE et al. *JAMA Network Open.*

Study Measure	Diagnosis Category	ICD-10 codes
		F14.920, F14.921, F14.922, F14.929, F14.94, F14.950, F14.951, F14.959, F14.980, F14.981, F14.982, F14.988, F14.99
	Other stimulant related disorders	F15.10, F15.120, F15.121, F15.122, F15.129, F15.14, F15.150, F15.151, F15.159, F15.180, F15.181, F15.182, F15.188, F15.19, F15.20, F15.21, F15.220, F15.221, F15.222, F15.229, F15.23, F15.24, F15.250, F15.251, F15.259, F15.280, F15.281, F15.282. F15.288, F15.29, F15.90, F15.920, F15.921, F15.922, F15.929 F15.93, F15.94, F15.950, F15.951, F15.959, F15.980, F15.981, F15.982, F15.988, F15.99
	Hallucinogen related disorders	F16.10, F16.120, F16.121, F16.122, F16.129, F16.14, F16.150, F16.151, F16.159, F16.180, F16.183, F16.188, F16.19, F16.20, F16.21, F16.220, F16.221, F16.229, F16.24, F16.250, F16.251, F16.259, F16.280, F16.283, F16.288, F16.29, F16.90, F16.920, F16.921, F16.929, F16.94, F16.950, F16.951, F16.959, F16.980, F16.983, F16.988, F16.99
	Inhalant related disorders	F18.10, F18.120, F18.121, F18.129, F18.14, F18.150, F18.151, F18.159, F18.17, F18.180, F18.188, F18.19, F18.20, F18.21, F18.220, F18.221, F18.229, F18.24, F18.250, F18.251, F18.259, F18.27, F18.280, F18.288, F18.29, F18.90, F18.920, F18.921, F18.929, F18.94, F18.950, F18.951, F18.959, F18.97, F18.980, F18.988, F18.99
	Other psychoactive substance related disorders	F19.10, F19.120, F19.121, F19.122, F19.129, F19.14, F19.150, F19.151, F19.159, F19.16, F19.17, F19.180, F19.181, F19.182 F19.188, F19.19, F19.20, F19.21, F19.220, F19.221, F19.222, F19.229, F19.230, F19.231, F19.232, F19.239, F19.24, F19.250, F19.251, F19.259, F19.26, F19.27, F19.280, F19.281, F19.282, F19.288, F19.29, F19.90, F19.920, F19.921, F19.922, F19.929, F19.930, F19.931, F19.932, F19.939, F19.94, F19.950, F19.951, F19.959, F19.96, F19.980, F19.981, F19.982, F19.988, F19.99
Mental Health	Other mental disorders due to known physiological condition	F06.0, F06.2, F06.30, F06.31, F06.32, F06.33, F06.34, F06.4

© 2024 Russell LE et al. *JAMA Network Open.*

Study Measure	Diagnosis Category	ICD-10 codes
Conditions	Schizophrenia, schizotypal, delusional, and other non-mood psychotic disorders	F20.0, F20.1, F20.2, F20.3, F20.5, F20.81, F20.89, F20.9, F21., F22., F23. , F24. , F25.0, F25.1 , F25.8 , F25.9 , F28., F29.
	Manic episodes	F30.4, F30.8, F30.9
	Bipolar disorder	F31.0, F31.10, F31.11, F31.12, F31.13, F31.2, F31.30, F31.31, F31.32, F31.4, F31.5, F31.60, F31.61, F31.62, F31.63, F31.64, F31.70, F31.71, F31.72, F31.73, F31.74, F31.75, F31.76, F31.77, F31.78, F31.81, F31.89, F31.9
	Depressive episode	F32.0, F32.1, F32.2, F32.3, F32.4, F32.5, F32.8, F32.9
	Major depressive disorder, recurrent	F33.0, F33.1, F33.2, F33.3, F33.40, F33.41, F33.42, F33.8, F33.9
	Persistent mood [affective] disorders	F34.0, F34.1, F34.8, F34.9
	Unspecified mood [affective] disorder	F39., F39.0
	Phobic anxiety disorders	F40., F40.0, F40.00, F40.01, F40.02, F40.10, F40.11, F40.210, F40.218, F40.220, F40.228, F40.230, F40.231, F40.232, F40.233, F40.240, F40.241, F40.242, F40.243, F40.248, F40.290, F40.291, F40.298, F40.8, F40.9
	Other anxiety disorders	F41., F41.0, F41.1, F41.3, F41.8, F41.9
	Obsessive-compulsive disorder	F42., F42.0
	Reaction to severe stress, and adjustment disorders	F43., F43.0, F43.10, F43.11, F43.12, F43.20, F43.21, F43.22, F43.23, F43.24, F43.25, F43.29, F43.8, F43.9
	Dissociative and conversion disorders	F44.0, F44., F44.1, F44.2, F44.4, F44.5, F44.6, F44.7, F44.81, F44.89, F44.9
	Somatoform disorders	F45.0, F45., F45.1, F45.20, F45.21, F45.22, F45.29, F45.8, F45.9
	Other nonpsychotic mental disorder	F48.1

© 2024 Russell LE et al. *JAMA Network Open.*

Study Measure	Diagnosis Category	ICD-10 codes
	Behavioral syndromes associated with physiological disturbances and physical factors	F50.00, F50., F50.0, F50.01, F50.02, F50.2, F50.8, F50.9
	Mental and behavioral disorders associated with the puerperium, not elsewhere classified	F53., F53.0
	Specific personality disorders	F60., F60.0, F60.1, F60.2, F60.3, F60.4, F60.5, F60.6, F60.7, F60.81, F60.89, F60.9
	Impulse disorders	F63.0, F63., F63.1, F63.2, F63.3, F63.81, F63.89, F63.9
	Other disorders of adult personality and behavior	F68.10, F68.11, F68.12, F68.13, F68.8
	Unspecified disorder of adult personality and behavior	F69., F69.0
	Attention-deficit hyperactivity disorders	F90., F90.0, F90.1, F90.2, F90.8, F90.9
	Conduct disorders	F91.0, F91.1, F91.2, F91.3, F91.8, F91.9

© 2024 Russell LE et al. *JAMA Network Open.*

eTable 2 Sociodemographic Correlates of Screening Positive for Not Having a Smart Phone or a Computer

	Unadjusted bivariate estimates			Adjusted multivariate model estimate[a]								
	OR	OR 95% CI (%)	OR P-Value	aOR	aOR CI (%)	aOR P-value	AP (%)	Adjusted prevalence[b] 95% CI (%)	AP P-value	Marginal effects (% pt)[c]	ME 95% CI (%)	dY/dX P-Value
Age 18-49							3.4	(2.1, 4.6)	<0.0001	ref		<0.001
Age 50-64	3.35	(2.22, 5.1)	<0.001	3.21	(2.08, 4.94)	<0.0001	9.9	(8.2, 11.6)	<0.0001	6.56	(4.5, 8.7)	<0.001
Age 65-79	6.57	(4.54, 9.73)	<0.001	6.42	(4.25, 9.69)	<0.0001	17.9	(16.5, 19.2)	<0.0001	14.48	(12.5, 16.4)	<0.001
Age 80+	14.89	(10.1, 22.1)	<0.001	13.49	(8.77, 20.75)	<0.0001	30.8	(27.9, 33.7)	<0.0001	27.41	(24.1, 30.7)	<0.001
Male	ref						17.6	(16.7, 18.5)	<0.0001	ref		<0.001
Female	0.19	(0.1, 0.3)	<0.001	0.38	(0.27, 0.54)	<0.0001	7.9	(5.5, 10.3)	<0.0001	-9.74	(-12.3, -7.2)	<0.001
White	ref						17.3	(16.2, 18.4)	<0.0001	ref		
Black or African American	0.77	(0.6, 0.9)	<0.001	0.89	(0.75, 1.06)	0.19	15.8	(14, 17.6)	<0.0001	-1.45	(-3.6, 0.7)	0.19
Other Race	0.80	(0.5, 1.2)	0.20	1.08	(0.69, 1.67)	0.74	18.3	(12.4, 24.2)	<0.0001	0.98	(-5, 7)	0.75
Unknown/Missing Race	0.73	(0.5, 1.0)	0.05	0.88	(0.55, 1.40)	0.59	15.7	(10.3, 21.1)	<0.0001	-1.61	(-7.3, 4)	0.58
Not Hispanic or Latino	ref						16.7	(15.8, 17.6)	<0.0001	ref		<0.001
Hispanic or Latino	0.87	(0.6, 1.3)	0.52	1.38	(0.90, 2.11)	0.14	21.1	(14.9, 27.4)	<0.0001	4.46	(-1.9, 10.8)	0.17
Unknown/Missing Ethnicity	0.78	(0.6, 11.)	0.14	1.16	(0.70, 1.91)	0.57	18.6	(11.9, 25.3)	<0.0001	1.93	(-5, 8.8)	0.58
Not Married/Partnered	ref						18.6	(17.2, 20.1)	<0.0001	ref		
Married/Partnered	0.72	(0.6, 0.8)	<0.001	0.78	(0.68, 0.90)	0.001	15.5	(14.3, 16.6)	<0.0001	-3.17	(-5, -1.3)	<0.001
Urban	ref						14.9	(13.7, 16)	<0.0001	ref		<0.001
Rural	1.46	(1.3, 1.7)	<0.001	1.45	(1.25, 1.68)	<0.001	19.7	(18.2, 21.2)	<0.0001	4.85	(2.9, 6.8)	<0.001
Unknown/Missing Rurality	0.65	(0.5, 0.9)	<0.005	1.09	(0.80, 1.49)	0.57	15.9	(12.3, 19.5)	<0.0001	1.07	(-2.7, 4.9)	0.58
Non-Service Connected & Low-Income	ref						20.8	(19, 22.7)	<0.0001	ref		
Service Connected	0.52	(0.4, 0.6)	<0.001	0.65	(0.55, 0.76)	<0.001	15.0	(13.8, 16.2)	<0.0001	-5.88	(-8.1, -3.6)	<0.001
Non-Service Connected	0.77	(0.6, 0.9)	<0.01	0.73	(0.60, 0.90)	0.003	16.5	(14.4, 18.6)	<0.0001	-4.35	(-7.1, -1.6)	0.002
Unknown/Missing VA Priority	0.14	(0.01, 1.0)	0.05	0.29	(0.04, 2.39)	0.25	7.7	(-6.6, 22)	0.2901	-13.12	(-27.5, 1.3)	0.07

OR=odds ratio, aOR=adjusted odds ratio, AP=predicted prevalence, ME=marginal effect

Notes: (a) Adjusted models include covariates in the table and clinical conditions (medically complex (CAN score 95+), smoking/tobacco use, diabetes, heart failure, hypertension, dementia, psychiatric diagnosis, substance use disorder).

(b) Model-adjusted prevalences are the population-average predictive margins, by subgroup, from a multivariate logistic regression.

(c) Marginal effects (ME) are calculated as the population-average percentage point change in prevalence associated with the subgroup, compared to the reference group.

© 2024 Russell LE et al. *JAMA Network Open.*

eTable 3. Sociodemographic Correlates of Screening Positive for Not Having Affordable or Reliable Internet

	Unadjusted bivariate estimates			Adjusted multivariate model estimate[(a)]								
	OR	OR 95% CI (%)	OR P-value	aOR	aOR 95% CI (%)	aOR P-value	Adjusted prevalence (AP) (%)	AP 95% CI (%)	AP P-value	Marginal effect (% pt)[(b)]	ME 95% CI (%)	dY/dX P-value
Age 18-49	Ref						15.0	(12.1, 18)	<0.01	ref		<0.001
Age 50-64	2.49	(2.0, 3.2)	<0.001	1.71	(1.31, 2.23)	<0.001	22.8	(20.5, 25.1)	<0.01	7.78	(4.1, 11.4)	<0.001
Age 65-79	2.83	(2.2 3.5)	<0.001	1.87	(1.44, 2.43)	<0.001	24.4	(22.9, 25.9)	<0.01	9.37	(5.9, 12.8)	<0.001
Age 80+	2.73	(2.1, 3.4)	<0.001	1.97	(1.47, 2.64)	<0.001	25.3	(22.6, 27.9)	<0.01	10.26	(6.1, 14.5)	<0.001
Male	Ref						23.9	(22.8, 25)	<0.01	ref		<0.001
Female	0.47	(0.4, 0.6)	<0.001	0.60	(0.48, 0.76)	<0.001	16.3	(13.4, 19.2)	<0.01	-7.65	(-10.8, -4.5)	<0.001
White	Ref						19.4	(18.2, 20.7)	<0.01	ref		
Black or African American	2.14	(1.9, 2.4)	<0.001	1.92	(1.67, 2.20)	<0.001	31.1	(28.9, 33.4)	<0.01	11.7	(9.1, 14.3)	<0.001
Other Race	1.70	(1.2, 2.3)	<0.001	2.01	(1.43, 2.83)	<0.001	32.1	(25.2, 39)	<0.01	12.64	(5.6, 19.7)	<0.001
Unknown/Missing Race	0.98	(0.6, 1.2)	0.35	1.08	(0.72, 1.63)	0.70	20.7	(14.5, 26.8)	<0.01	1.24	(-5.2, 7.7)	0.70
Not Hispanic or Latino	Ref						23.3	(22.2, 24.3)	<0.01	ref		
Hispanic or Latino	0.62	(0.4, 0.9)	0.001	0.82	(0.55, 1.23)	0.34	20.1	(14.1, 26.1)	<0.01	-3.15	(-9.3, 3.0)	0.31
Unknown/Missing Ethnicity	0.77	(0.5, 0.8)	0.001	0.97	(0.63, 1.51)	0.90	22.8	(15.7, 29.8)	<0.01	-0.47	(-7.8, 6.8)	0.90
Not Married/Partnered	Ref						25.2	(23.6, 26.8)	<0.01	ref		<0.001
Married/Partnered	0.73	(0.6, 0.8)	<0.001	0.81	(0.71, 0.91)	0.007	21.6	(20.2, 22.9)	<0.01	-3.61	(-5.7, -1.5)	<0.001
Urban	Ref						23.8	(22.4, 25.2)	<0.01	ref		<0.001
Rural	0.78	(0.7, 0.9)	<0.001	0.97	(0.85, 1.11)	0.68	23.3	(21.6, 25.2)	<0.01	-0.47	(-27.4, 1.8)	0.68
Unknown/Missing Rurality	0.39	(0.3, 0.5)	<0.001	0.65	(0.49, 0.86)	0.003	17.2	(13.5, 20.8)	<0.01	-6.64	(-10.6, -2.7)	<0.001
Non-Service Connected & Low-Income	Ref		<0.001				27.4	(25.3, 29.5)	<0.01	ref		<0.001
Service Connected	0.66	(0.6, 0.8)	<0.001	0.74	(0.65, 0.86)	<0.001	22.2	(20.9, 23.5)	<0.01	-5.19	(-7.8, -2.6)	<0.001
Non-Service Connected	0.51	(0.5, 0.7)	<0.001	0.63	(0.52, 0.77)	<0.001	19.7	(17.2, 22.1)	<0.01	-7.73	(-11.0, -4.5)	<0.001
Unknown/Missing VA Priority	0.38	(0.2, 1.3)	0.13	0.99	(0.32, 3.01)	0.98	27.1	(6.4, 47.9)	0.0105	-0.28	(-21.1, 20.6)	0.98

OR=odds ratio, aOR=adjusted odds ratio, AP=predicted prevalence, ME=marginal effect

Notes: (a) Adjusted models include clinical conditions (medically complex (CAN score 95+), smoking/tobacco use, diabetes, heart failure, hypertension, dementia, psychiatric diagnosis, substance use disorder). (b) Marginal effects (ME) are calculated as the average percentage point change associated with having the covariate, vs. not having the covariate.

© 2024 Russell LE et al. *JAMA Network Open.*

eTable 4. Sociodemographic Correlates of Screening Positive for Running Out of Minutes Sometimes or Often

	Unadjusted bivariate estimates			Adjusted multivariate model estimate[(a)]								
	OR	OR 95% CI (%)	OR P-value	aOR	aOR 95% CI	aOR P-value	Predicted prevalence (%)	PP 95% CI (%)	PP P-value	Marginal effects (% pt)[(b)]	ME 95% CI (%)	dY/dX P-value
Age 18-49							5.8	(3.6, 7.9)	<0.01	ref		<0.001
Age 50-64	1.08	(0.7, 1.5)	0.90	0.64	(0.40, 1.04)	0.07	3.8	(2.8, 4.8)	<0.01	-1.93	(-4.3, 0.4)	0.10
Age 65-79	0.55	(0.4, 0.8)	<0.001	0.39	(0.24, 0.64)	0.0002	2.4	(1.8, 2.9)	<0.01	-3.39	(-5.7, -1.1)	0.004
Age 80+	0.2	(0.1, 0.4)	<0.001	0.19	(0.09, 0.40)	<0.0001	1.2	(0.5, 1.8)	<0.01	-4.61	(-7.0, -2.3)	0.01
Male	Ref						2.8	(2.4, 3.2)	<0.01	ref		<0.001
Female	1.65	(1.1, 2.5)	<0.001	1.31	(0.86, 1.99)	0.20	3.6	(2.3, 4.9)	<0.01	0.82	(-0.6, 2.2)	0.24
White	Ref						2.4	(1.9, 2.9)	<0.01	ref		<0.001
Black or African American	1.81	(1.4, 2.6)	<0.001	1.56	(1.11, 2.18)	0.01	3.7	(2.8, 4.6)	<0.01	1.27	(0.2, 2.3)	0.02
Other Race	0.93	(0.3, 26)	0.89	0.78	(0.28, 2.17)	0.63	1.9	(0.1, 3.8)	0.04	-0.52	(-2.4, 1.4)	0.60
Unknown/Missing Race	1.74	(0.9, 3.0)	0.08	2.16	(1.02, 4.57)	0.04	5.0	(1.7, 8.3)	<0.01	2.57	(-0.8, 5.9)	0.13
Not Hispanic or Latino	Ref						3.0	(2.5, 3.4)	<0.01	ref		<0.001
Hispanic or Latino	1.97	(1.0, 3.7)	0.04	1.62	(0.83, 3.18)	0.16	4.6	(1.9, 7.4)	<0.01	1.68	(-1.1, 4.5)	0.24
Unknown/Missing Ethnicity	0.83	(0.4, 1.7)	0.53	0.49	(0.18, 1.31)	0.15	1.5	(0.1, 2.8)	0.03	-1.48	(-3.0, 0.04)	0.06
Not Married/Partnered	Ref						3.1	(2.4, 3.7)	<0.01	ref		<0.001
Married/Partnered	0.77	(0.6, 1.0)	0.08	0.91	(0.67, 1.24)	0.56	2.8	(2.3, 3.4)	<0.01	-0.25	(-1.1, 0.6)	0.56
Urban	Ref						3.3	(2.7, 3.9)	<0.01	ref		<0.001
Rural	0.71	(0.5, 0.9)	0.01	0.84	(0.60, 1.19)	0.32	2.8	(2, 3.5)	<0.01	-0.49	(-1.5, 0.5)	0.31
Unknown/Missing Rurality	0.56	(0.3, 1.0)	0.05	0.47	(0.25, 0.89)	0.02	1.6	(0.7, 2.5)	<0.01	-1.69	(-2.8, -0.6)	<0.001
Non-Service Connected & Low-Income	Ref						4.9	(3.7, 6)	<0.01	ref		<0.001
Service Connected	0.61	(0.4, 0.8)	<0.001	0.50	(0.36, 0.69)	<0.001	2.5	(2, 3)	<0.01	-2.34	(-3.6, -1.1)	<0.001
Non-Service Connected	0.33	(0.1, 0.5)	<0.001	0.30	(0.17, 0.56)	<0.001	1.6	(0.7, 2.4)	<0.01	-3.29	(-4.7, -1.9)	<0.001
Unknown/Missing VA Priority	-	(0, Inf)	0.97	0.00	(0.00, Inf)	0.98	0.0	(-0.01, 0.01)	0.99	-485	(-6.0, -3.7)	<0.001

Notes: (a) Adjusted models include clinical conditions (medically complex (CAN score 95+), smoking/tobacco use, diabetes, heart failure, hypertension, dementia, psychiatric diagnosis, substance use disorder). (b) Marginal effects (ME) are calculated as the average percentage point change associated with having the covariate, vs. not having the covariate.

© 2024 Russell LE et al. *JAMA Network Open.*

eTable 5. Sociodemographic Correlates of Screening Positive for Requesting Assistance in Setting Up a Video Telehealth Encounter

	Unadjusted bivariate estimates			Adjusted multivariate model estimate[(a)]								
	OR	OR 95% CI	OR P-value	aOR	aOR 95% CI	aOR P-value	Adjusted prevalence (AP) (%)	AP 95% CI (%)	AP P-value	Marginal effects (%)	Marginal effects 95% CI (%)	dY/dX P-value
Age 18-49							10.5	(8.2, 12.9)	<0.001	ref		<0.001
Age 50-64	1.35	(1.09, 1.84)	0.01	1.39	(1.03, 1.86)	0.03	14.0	(12, 15.9)	<0.001	3.45	(0.5, 6.4)	0.02
Age 65-79	1.07	(0.87, 1.39)	0.44	1.10	(0.82, 1.48)	0.52	11.5	(10.3, 12.6)	<0.01	0.93	(-1.8, 3.7)	0.51
Age 80+	1.19	(0.94, 1.63)	0.12	1.29	(0.92, 1.81)	0.15	13.1	(11, 15.3)	<0.001	2.58	(-0.9, 6.0)	0.14
Male							12.4	(11.6, 13.3)	<0.001	ref		
Female	0.79	(0.59, 0.99)	0.04	0.74	(0.56, 0.97)	0.03	9.5	(7.3, 11.7)	<0.001	-2.89	(-5.3, -0.5)	0.02
White							11.0	(10.1, 12)	<0.001	ref		<0.001
Black or African American	1.27	(1.06, 1.48)	<0.001	1.22	(1.02, 1.47)	0.03	13.2	(11.5, 14.8)	<0.001	2.12	(0.2, 4.1)	0.03
Other Race	1.90	(1.32, 2.81)	<0.001	1.97	(1.34, 2.89)	<0.001	19.5	(13.8, 25.3)	<0.001	8.47	(2.7, 14.3)	<0.001
Unknown/Missing Race	1.23	(0.90, 1.69)	0.19	1.52	(0.98, 2.37)	0.06	15.8	(10.3, 21.4)	<0.001	4.8	(-0.9, 10.5)	0.10
Not Hispanic or Latino							12.2	(11.4, 13.1)	<0.001	ref		
Hispanic or Latino	1.17	(0.81, 1.82)	0.33	1.20	(0.79, 1.82)	0.40	14.2	(9.4, 19.1)	<0.001	2.01	(-3.0, 7.0)	0.43
Unknown/Missing Ethnicity	0.88	(0.62, 1.29)	0.54	0.71	(0.43, 1.18)	0.18	9.0	(5.1, 13)	<0.001	-3.21	(-7.4, 1.0)	0.13
Not Married/Partnered							12.9	(11.6, 14.1)	<0.001	ref		
Married/Partnered	0.82	(0.72, 0.97)	0.02	0.88	(0.76, 1.03)	0.13	11.6	(10.5, 12.6)	<0.001	-1.3	(-3.0, 0.4)	0.13
Urban							11.8	(10.7, 12.9)	<0.001	ref		
Rural	1.01	(0.85, 1.17)	0.93	1.07	(0.91, 1.27)	0.41	12.6	(11.2, 14)	<0.001	0.75	(-1.1, 2.6)	0.41
Unknown/Missing Rurality	0.85	(0.70, 1.20)	0.52	1.01	(0.74, 1.36)	0.96	11.9	(9.0, 14.8)	<0.001	0.07	(-3.1, 3.2)	0.96
Non-Service Connected & Low-Income							13.9	(12.2, 15.6)	<0.001	ref		
Service Connected	0.82	(0.67, 0.95)	0.001	0.84	(0.70, 1.00)	0.05	11.9	(10.9, 13.0)	<0.001	-1.98	(-4.0, 0.1)	0.06
Non-Service Connected	0.60	(0.47, 0.78)	<0.001	0.66	(0.51, 0.86)	<0.01	9.7	(7.9, 11.6)	<0.001	-4.19	(-6.7, -1.7)	0.001
Unknown/Missing VA Priority	1.12	(0.37, 3.15)	0.89	1.12	(0.37, 3.41)	0.84	15.3	(1.2, 29.5)	0.03	1.4	(-12.9, 15.6)	0.85

Notes: (a) Adjusted models include clinical conditions (medically complex (CAN score 95+), smoking/tobacco use, diabetes, heart failure, hypertension, dementia, psychiatric diagnosis, substance use disorder). (b) Marginal effects (ME) are calculated as the average percentage point change associated with having the covariate, vs. not having the covariate.

© 2024 Russell LE et al. *JAMA Network Open.*

eTable 6. Logistic Model Predicted Margins and 95% Confidence Intervals (Without smartphone or computer; without affordable and reliable internet)

	Without smartphone or computer						Without affordable and reliable internet					
	Unadjusted			Adjusted[a]			Unadjusted			Adjusted[a]		
	Prevalence	CI lower	CI upper	Adjusted prevalence	CI lower	CI upper	Prevalence	CI lower	CI upper	Adjusted prevalence	CI lower	CI upper
Ages 18-49	0.032	0.020	0.043	0.034	0.021	0.046	0.111	0.091	0.131	0.150	0.121	0.180
Ages 50-64	0.098	0.082	0.115	0.099	0.082	0.116	0.237	0.213	0.261	0.228	0.205	0.251
Ages 65-79	0.178	0.164	0.191	0.179	0.165	0.192	0.258	0.242	0.273	0.244	0.229	0.259
Ages 80+	0.327	0.300	0.354	0.308	0.279	0.337	0.252	0.227	0.276	0.253	0.226	0.279
Male	0.183	0.173	0.193	0.176	0.167	0.186	0.243	0.232	0.254	0.239	0.228	0.250
Female	0.052	0.035	0.068	0.079	0.055	0.103	0.141	0.116	0.167	0.163	0.134	0.192
White	0.183	0.171	0.194	0.173	0.162	0.184	0.191	0.179	0.203	0.194	0.182	0.206
Black or African American	0.144	0.127	0.160	0.158	0.140	0.176	0.334	0.312	0.356	0.311	0.289	0.334
Other Race	0.145	0.095	0.196	0.183	0.124	0.242	0.285	0.220	0.350	0.321	0.252	0.390
Unknown/Missing Race	0.141	0.106	0.177	0.157	0.103	0.211	0.171	0.133	0.210	0.207	0.145	0.268
Not Hispanic or Latino	0.171	0.161	0.180	0.167	0.158	0.176	0.237	0.226	0.248	0.233	0.222	0.243
Hispanic or Latino	0.153	0.104	0.203	0.211	0.149	0.274	0.158	0.108	0.209	0.201	0.141	0.261
Unknown/Missing Ethnicity	0.138	0.100	0.177	0.186	0.119	0.253	0.170	0.129	0.212	0.228	0.157	0.298
Not MarriedPartnered	0.194	0.179	0.209	0.186	0.172	0.201	0.272	0.255	0.288	0.252	0.236	0.267
MarriedPartnered	0.150	0.138	0.161	0.155	0.143	0.166	0.202	0.190	0.215	0.215	0.202	0.229
Urban	0.150	0.138	0.162	0.148	0.137	0.160	0.260	0.246	0.275	0.238	0.224	0.252
Rural	0.212	0.195	0.228	0.197	0.182	0.212	0.219	0.203	0.236	0.233	0.216	0.251
Missing Rurality	0.107	0.082	0.131	0.159	0.123	0.195	0.121	0.095	0.147	0.172	0.135	0.208
VA_Priority: Non-Service Connected & Low-Income	0.236	0.215	0.257	0.208	0.190	0.227	0.293	0.271	0.316	0.274	0.252	0.295
VA_Priority: Service Connected	0.134	0.123	0.145	0.150	0.138	0.161	0.219	0.206	0.232	0.222	0.208	0.235
VA_Priority: Non-Service Connected	0.193	0.169	0.217	0.165	0.144	0.186	0.186	0.162	0.209	0.197	0.172	0.221
VA_Priority: Unknown/Missing	0.038	-0.035	0.112	0.077	-0.066	0.220	0.154	0.015	0.293	0.271	0.064	0.479

(a) Model-adjusted prevalences are the population-average predictive margins, by stratum, from a logistic regression model adjusting for other observed characteristics in the table and clinical conditions (medically complex (CAN score 95+), smoking/tobacco use, diabetes, heart failure, hypertension, dementia, psychiatric diagnosis, substance use disorder.

© 2024 Russell LE et al. *JAMA Network Open.*

eTable 7. Logistic Model Predicted Margins and 95% Confidence Intervals (Run out of phone minutes or data; want help with a video or telehealth visit)

	Run out of phone minutes or data						Want help with a video telehealth visit					
	Unadjusted			Adjusted[(a)]			Unadjusted			Adjusted[(a)]		
	Prevalence	CI lower	CI upper	Adjusted prevalence	CI lower	CI upper	Prevalence	CI lower	CI upper	Adjusted prevalence	CI lower	CI upper
Ages 18-49	0.046	0.032	0.059	0.058	0.036	0.079	0.105	0.086	0.125	0.105	0.082	0.129
Ages 50-64	0.047	0.035	0.059	0.038	0.028	0.048	0.143	0.123	0.162	0.140	0.120	0.159
Ages 65-79	0.025	0.019	0.030	0.024	0.018	0.029	0.115	0.103	0.126	0.115	0.103	0.126
Ages 80+	0.009	0.004	0.015	0.011	0.005	0.018	0.127	0.108	0.147	0.131	0.110	0.153
Male	0.027	0.023	0.031	0.028	0.024	0.032	0.124	0.115	0.133	0.124	0.116	0.133
Female	0.045	0.030	0.060	0.036	0.023	0.049	0.098	0.076	0.120	0.095	0.073	0.117
White	0.023	0.018	0.028	0.024	0.019	0.029	0.111	0.101	0.120	0.110	0.101	0.120
Black or African American	0.043	0.033	0.052	0.037	0.028	0.046	0.135	0.119	0.151	0.132	0.115	0.148
Other Race	0.022	0.001	0.042	0.019	0.001	0.038	0.194	0.137	0.250	0.195	0.138	0.252
Unknown/Missing Race	0.038	0.018	0.058	0.050	0.017	0.083	0.133	0.098	0.168	0.158	0.103	0.214
Not Hispanic or Latino	0.029	0.024	0.033	0.030	0.025	0.034	0.121	0.113	0.129	0.122	0.114	0.131
Hispanic or Latino	0.054	0.023	0.086	0.046	0.019	0.074	0.144	0.095	0.192	0.142	0.093	0.191
Unknown/Missing Ethnicity	0.023	0.006	0.039	0.015	0.001	0.028	0.109	0.075	0.144	0.090	0.051	0.130
Not MarriedPartnered	0.033	0.027	0.040	0.031	0.024	0.037	0.132	0.119	0.145	0.128	0.116	0.141
MarriedPartnered	0.026	0.021	0.031	0.028	0.022	0.033	0.113	0.103	0.123	0.115	0.105	0.126
Urban	0.035	0.029	0.041	0.033	0.027	0.039	0.122	0.111	0.133	0.118	0.107	0.129
Rural	0.023	0.017	0.029	0.028	0.020	0.035	0.121	0.108	0.135	0.126	0.112	0.140
Missing Rurality	0.019	0.009	0.030	0.016	0.006	0.025	0.113	0.088	0.138	0.119	0.090	0.148
VA_Priority: Non-Service Connected & Low-Income	0.045	0.034	0.055	0.049	0.037	0.060	0.144	0.127	0.162	0.139	0.122	0.156
VA_Priority: Service Connected	0.028	0.022	0.033	0.025	0.020	0.030	0.119	0.109	0.129	0.119	0.109	0.130
VA_Priority: Non-Service Connected	0.012	0.006	0.019	0.016	0.007	0.024	0.093	0.075	0.110	0.097	0.079	0.116
VA_Priority: Unknown/Missing	0.000	0.000	0.000	0.000	0.000	0.000	0.154	0.015	0.293	0.153	0.012	0.295

((a) Model-adjusted prevalences are the population-average predictive margins, by stratum, from a logistic regression model adjusting for other observed characteristics in the table and clinical conditions (medically complex (CAN score 95+), smoking/tobacco use, diabetes, heart failure, hypertension, dementia, psychiatric diagnosis, substance use disorder.

© 2024 Russell LE et al. *JAMA Network Open.*

eTable 8. Poisson models of association of clinical conditions and being without smartphone or computer

Poisson Model: Without smartphone or computer

	Unadjusted				Adjusted[(a)]			
variable	RR	CI lower	CI upper	Pr(>|z|)	RR	CI lower	CI upper	Pr(>|z|)
High risk (CAN score 95+)	1.09	0.97	1.24	0.14	0.94	0.81	1.10	0.45
Smoking/tobacco use	0.88	0.75	1.03	0.11	0.89	0.75	1.05	0.17
Diabetes	0.99	0.87	1.12	0.89	0.91	0.79	1.04	0.16
Heart failure	1.11	0.96	1.29	0.17	0.98	0.83	1.16	0.82
Hypertension	1.26	1.10	1.43	<0.01	0.86	0.74	0.99	0.035
Dementia	1.71	1.42	2.06	<0.01	1.21	1.00	1.48	0.045
Psychiatric diagnosis	0.71	0.62	0.80	<0.01	0.98	0.85	1.12	0.71
Substance use disorder	0.94	0.80	1.11	0.46	1.33	1.10	1.60	<0.001

Notes: (a) Adjusted model includes variables in the table and age, sex, race, Hispanic ethnicity, rurality, and VHA priority enrollment.

© 2024 Russell LE et al. *JAMA Network Open.*

eTable 9. Poisson models of association of clinical conditions and being without affordable and reliable internet

Poisson Model: Without affordable and reliable internet

	Unadjusted				Adjusted[(a)]			
variable	RR	CI lower	CI upper	Pr(>|z|)	RR	CI lower	CI upper	Pr(>|z|)
High risk (CAN score 95+)	1.57	1.42	1.74	<0.001	1.26	1.11	1.42	<0.001
Smoking/tobacco use	1.33	1.18	1.49	<0.001	1.15	1.01	1.31	0.042
Diabetes	1.09	0.98	1.21	0.11	0.91	0.81	1.02	0.12
Heart failure	1.28	1.13	1.45	<0.001	0.99	0.86	1.13	0.84
Hypertension	1.31	1.18	1.47	<0.001	0.98	0.86	1.12	0.77
Dementia	1.00	0.82	1.22	0.99	0.85	0.69	1.04	0.11
Psychiatric diagnosis	0.90	0.81	1.00	0.05	0.91	0.81	1.02	0.10
Substance use disorder	0.94	0.80	1.11	0.46	1.05	0.91	1.22	0.51

Notes: (a) Adjusted model includes variables in the table and age, sex, race, Hispanic ethnicity, rurality, and VHA priority enrollment.

© 2024 Russell LE et al. *JAMA Network Open.*

eTable 10. Poisson models of association of clinical conditions and running out of phone minutes or data

Poisson Model: Run out of phone minutes or data								
	Unadjusted				Adjusted[a]			
variable	RR	CI lower	CI upper	Pr(>|z|)	RR	CI lower	CI upper	Pr(>|z|)
High risk (CAN score 95+)	1.07	0.80	1.44	0.65	1.13	0.80	1.61	0.49
Smoking/tobacco use	1.33	0.96	1.88	0.09	1.16	0.80	1.68	0.42
Diabetes	0.78	0.57	1.07	0.13	0.99	0.70	1.40	0.95
Heart failure	0.45	0.28	0.75	0.002	0.47	0.27	0.80	<0.001
Hypertension	0.86	0.64	1.15	0.29	1.29	0.90	1.84	0.17
Dementia	0.50	0.24	1.06	0.07	0.70	0.32	1.52	0.37
Psychiatric diagnosis	1.56	1.17	2.08	0.002	1.03	0.74	1.42	0.88
Substance use disorder	2.45	1.80	3.33	<0.001	1.57	1.10	2.25	0.01

Notes: (a) Adjusted model includes variables in the table and age, sex, race, Hispanic ethnicity, rurality, and VHA priority enrollment.

© 2024 Russell LE et al. *JAMA Network Open.*

eTable 11. Poisson models of association of clinical conditions and wanting help with a video telehealth visit

	Poisson Model: Want help with a video telehealth visit							
	Unadjusted				Adjusted			
variable	RR	CI lower	CI upper	Pr(>|z|)	RR	CI lower	CI upper	Pr(>|z|)
High risk (CAN score 95+)	1.08	0.93	1.25	0.30	0.96	0.80	1.14	0.61
Smoking/tobacco use	1.16	0.98	1.38	0.08	1.11	0.92	1.34	0.29
Diabetes	0.97	0.84	1.13	0.70	1.00	0.85	1.18	0.98
Heart failure	0.93	0.77	1.12	0.42	0.90	0.73	1.11	0.34
Hypertension	0.98	0.85	1.13	0.76	0.92	0.76	1.10	0.36
Dementia	1.56	1.24	1.96	<0.001	1.58	1.24	2.01	<0.001
Psychiatric diagnosis	1.13	0.98	1.30	0.10	1.08	0.92	1.27	0.33
Substance use disorder	1.36	1.14	1.61	0.001	1.22	1.00	1.49	0.05

Notes: (a) Adjusted model includes variables in the table and age, sex, race, Hispanic ethnicity, rurality, and VHA priority enrollment.

© 2024 Russell LE et al. *JAMA Network Open.*
