# Supplementary material for: Sociodemographic and Clinical Characteristics Associated With Veterans’ Digital Needs
Source: JAMA Netw Open. 2024 Nov 15;7(11):e2445327. doi: 10.1001/jamanetworkopen.2024.45327 (PMC11568462; doi:10.1001/jamanetworkopen.2024.45327)
Supplement: Supplement 2. — Data Sharing Statement [file jamanetwopen-e2445327-s002.pdf]

Data Sharing Statement

Russell. Demographic and Clinical Characteristics Associated With Veterans' Digital Needs. *JAMA Netw Open*. Published November 15, 2024. doi:10.1001/jamanetworkopen.2024.45327

Data
Data available: No

Additional Information
Explanation for why data not available: We can provide a data dictionary and/or definitions for any included variables for our analyses, though we will not be able to include individual patient data, even if blinded or anonymized, due to privacy concerns.
